# Hypercalcemia as an Immune-Related Adverse Event in a Patient Receiving Nivolumab and Ipilimumab for Metastatic Melanoma: A Case Report

**DOI:** 10.1155/crom/8600200

**Published:** 2025-07-10

**Authors:** Sam Plessers, Jeroen Mebis, Nina De Moor, Tim Wessels, Daisy Luyten, Annelies Requilé

**Affiliations:** Department of Medical Oncology, Jessa Ziekenhuis, Hasselt, Belgium

**Keywords:** case report, hypercalcemia, immune checkpoint inhibition, malignant melanoma, sarcoid-like reaction

## Abstract

Hypercalcemia of malignancy is a well-known phenomenon in cancer patients, often associated with poor prognosis. The discovery of immune checkpoint inhibitors has revolutionised cancer therapy by improving prognosis in numerous different cancer types. Unfortunately, immune-related adverse events frequently arise, particularly with dual checkpoint inhibition. We present a case of severe hypercalcemia in a 65-year-old woman undergoing treatment for metastasised malignant melanoma. Eleven weeks after initiating ipilimumab–nivolumab, the patient developed severe hypercalcemia, along with inflammation and hepatitis. This was initially presumed to be due to hypercalcemia of malignancy, given the clinical examination, imaging findings and laboratory values potentially consistent with progressive disease. The hypercalcemia responded well to bisphosphonates, intravenous saline and methylprednisolone. Interestingly, fluorodeoxyglucose–positron emission tomography/computed tomography (FDG-PET/CT) performed shortly after hospital discharge showed a complete metabolic remission, thereby making hypercalcemia of malignancy unlikely. Review of her medical history, imaging and laboratory revealed several features consistent with a sarcoid-like reaction. We hypothesise that this reaction led to elevated 1-alpha hydroxylase, thereby facilitating calcitriol-mediated hypercalcemia. In this report, we summarise previously published case reports on immune checkpoint inhibitor-induced hypercalcemia and discuss the various mechanisms that cause hypercalcemia in this rare immune-related adverse event. Immune checkpoint inhibitors can induce production of parathyroid hormone–related protein (PTHrP), calcitriol, and may cause hypocortisolaemia, all of which can disrupt calcium homeostasis. Through this case, we contribute to the growing body of evidence regarding hypercalcemia as an immune-related adverse event and aim to raise awareness among clinicians of this potential complication. Early recognition is critical for this life-threatening condition, as it can be refractory to conventional therapies and may necessitate corticosteroid therapy.

## 1. Introduction

Hypercalcemia of malignancy is a well-known and common phenomenon, affecting approximately 20% of cancer patients during the course of their disease [1]. It typically presents with severe symptoms, requiring urgent treatment. Unfortunately, it is also associated with a very poor prognosis, illustrated by a recent retrospective analysis reporting a median overall survival of only 40 days [2]. Several mechanisms can contribute to the development of hypercalcemia in patients with cancer. Possible causes are the production of parathyroid hormone (PTH)–related peptide (PTHrP), bone metastases, calcitriol overproduction, or excess formation of ectopic PTH [3].

The discovery of immune checkpoint inhibitors has revolutionised cancer therapy and improved survival in numerous cancer types; however, their use can be complicated by immune-related adverse events. These events can lead to significant morbidity and, in rare cases, mortality [4]. The frequency of Grades 3–4 adverse events is particularly high with dual checkpoint inhibition, occurring in 35%–65% of patients treated with ipilimumab–nivolumab, depending on the dose [5]. In contrast with hypercalcemia of malignancy, hypercalcemia as an immune-related adverse event is exceedingly rare, and available evidence is mainly limited to a handful of case reports.

We present a case of severe hypercalcemia in a patient with metastatic melanoma treated with ipilimumab–nivolumab, not due to the malignancy but rather as an immune-related adverse event. In this case, the correct diagnosis was only made after patient discharge, underlining the diagnostic challenge and the risks associated with delayed recognition of this rare adverse event.

## 2. Case Presentation

In June 2020, a 61-year-old woman was diagnosed with superficial spreading malignant melanoma on the right ankle. Following surgical resection and sentinel lymph node biopsy, the tumour was classified as pT4bN1 according to the 8^th^ edition of the TNM staging system, corresponding to stage IIIC. The tumour harboured a gain-of-function mutation of v-raf murine sarcoma viral oncogene homolog B1 (BRAF) V600E. Fluorodeoxyglucose-positron emission tomography/computed tomography (FDG-PET/CT) revealed no distant metastases. During her adjuvant treatment with pembrolizumab, she developed multiple cutaneous metastases on the right lower leg and a new inguinal adenopathy after 3 months of therapy. Considered as inoperable, she started treatment with dabrafenib–trametinib, which led to rapid complete remission.

**Figure 1:**
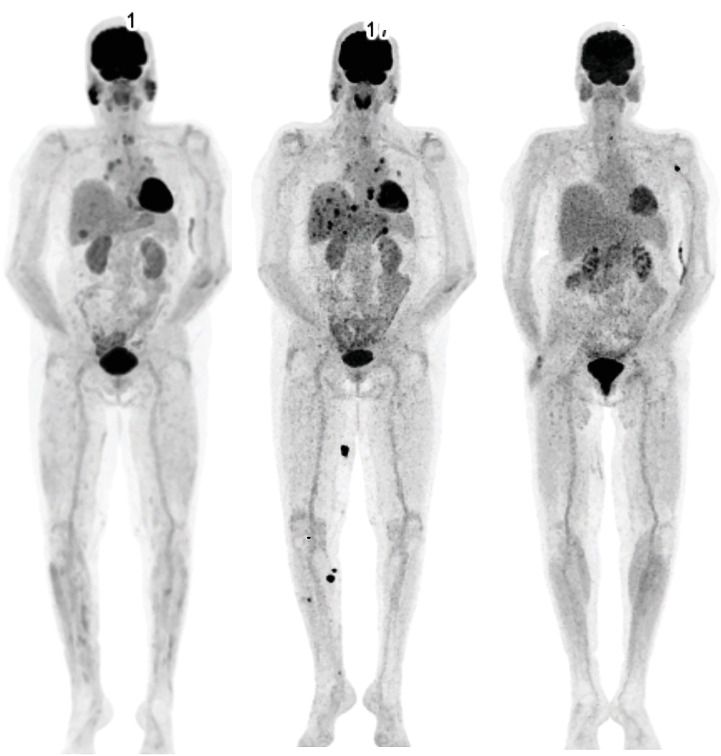
(a) FDG-PET from March 2024. (b) FDG-PET from August 2024. (c) FDG-PET from November 2024, following hospital discharge. (a, b) Note the persistent hypermetabolism in the mediastinum on the right side, corresponding to mediastinal and hilar lymphadenopathies due to a sarcoid-like reaction. (b) Multiple hypermetabolic metastases are evident; (c) complete metabolic remission.

Three months after initiation of dabrafenib–trametinib, FDG-PET/CT showed new moderately hypermetabolic mediastinal and hilar lymphadenopathies. Histological analysis via bronchoscopy showed multiple epithelioid histiocytes, consistent with a sarcoid-like reaction. The patient was asymptomatic, and treatment with steroids was not deemed necessary. The disease remained in complete remission, but dabrafenib–trametinib was discontinued after 3 years due to an acute kidney injury. After careful exclusion of other causes, the acute kidney injury was presumed to be induced by dabrafenib–trametinib. Although a kidney biopsy was recommended for further confirmation, the patient declined this option. Given the sustained remission of the disease over the 3-year period, a decision was made to pause the targeted therapy. Eight months later, in August 2024, the disease recurred. FDG-PET/CT revealed multiple lymphadenopathies, along with metastases in skin, pancreas, lung and predominantly the liver (Figure 1). Histology of a skin lesion confirmed metastasis of the BRAF-mutated malignant melanoma. Treatment with ipilimumab (3 mg/kg)–nivolumab (1 mg/kg) was initiated. At this time and during the preceding 3 years, her serum calcium levels had remained normal.

Ten weeks later, in November 2024, she presented at our day hospital for her fourth cycle of ipilimumab–nivolumab. She reported feeling generally well, but laboratory studies revealed new abnormalities (Table 1). Serum creatinine increased to 1.56 mg/dL (from a baseline of 1.09 mg/dL, reference range 0.81–0.95 mg/dL), and albumin-corrected serum calcium rose to 2.79 mmol/L (reference range 2.20–2.55 mmol/L). An increase in fluid intake was recommended, ipilimumab–nivolumab treatment continued, and the patient was discharged home.

The patient returned 1 week later with worsening symptoms, including muscle weakness, anorexia, confusion and sleepiness. On examination, she was normotensive, with a blood pressure of 101/72 mmHg. Peripheral pulse oximetry showed a saturation of 94%, and her heart rate was 90 beats per minute. Clinically, she was somnolent, disoriented, unable to stand up, and exhibited signs of mild dehydration. Palpation of the right upper abdominal quadrant elicited pain. Laboratory studies revealed a significant increase in albumin-corrected serum calcium to 3.84 mmol/L, a serum creatinine of 1.72 mg/dL, a CRP of 92 mg/L (reference range < 5 mg/L), and elevated transaminase levels, which narrowly exceeded five times the upper limit of normal. PTH was suppressed at 8.4 ng/L (reference range 15–68 ng/L), while 25-OH vitamin D was within the normal range at 32.5 *μ*g/dL. Angiotensin converting enzyme (ACE) was elevated above 150 U/L (reference range 8–52 U/L), but the result was not available until 1 week later. Viral serology did not indicate viral hepatitis. PTHrP and calcitriol were not measured, as the results would take several weeks to obtain in our centre. Given the patient's clinical presentation and laboratory values, we suspected progression of metastatic malignant melanoma, and a tentative diagnosis of hypercalcemia of malignancy was made. She was admitted to the hospital and treated with intravenous pamidronate (90 mg) and isotonic saline.

**Figure 2:**
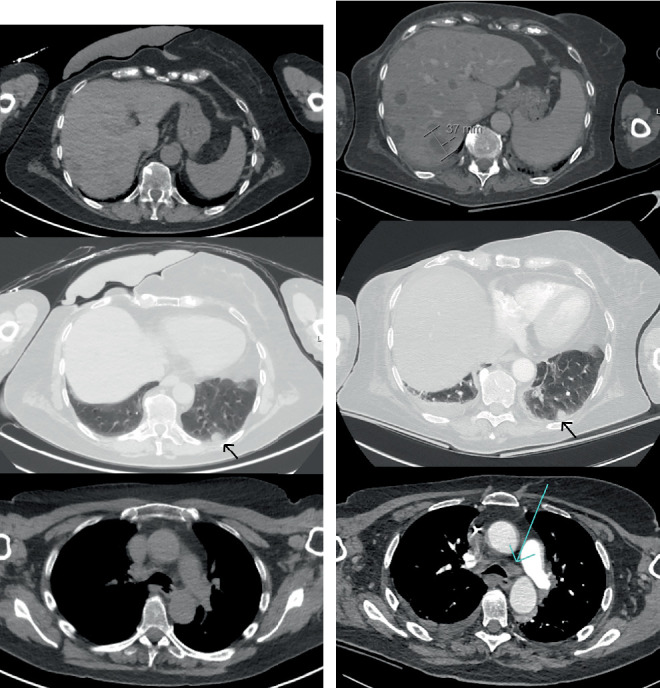
(a) CT images from FDG-PET/CT in August 2024. (b) CT during admission in November 2024; multiple liver metastases are visible, with the largest lesion measuring 37 mm. A stable lung metastasis is indicated by the black arrow, and a new mediastinal lymphadenopathy is seen in Region 4L, marked by the blue arrow.

Methylprednisolone (32 mg) was administered on Day 2 as prophylaxis due to suspected iodine contrast allergy. Magnetic resonance imaging (MRI) of the brain revealed no metastases or ischaemia. A CT scan of the chest and upper abdomen was compared to the FDG-PET/CT from August 2024. There were no bone metastases, but a slight increase in hepatomegaly and multiple liver metastases was observed. Previous imaging had not shown such extensive liver metastases, possibly due to the omission of intravenous contrast, which hindered accurate interpretation. Two lung lesions remained stable. A small peripheral lung embolism, mild pleural effusion and newly enlarged mediastinal lymphadenopathies were identified (Figure 2). Although the imaging needed to be interpreted cautiously, it was potentially consistent with progressive disease in the liver and mediastinum. She was initiated on a therapeutic dose of low-molecular-weight heparin.

On Day 2 of admission, she developed a fever and was started on intravenous piperacillin–tazobactam (4 g every 4 h). Serum calcium levels normalised completely by Day 5. Despite continued antibiotic therapy, fever persisted and serum CRP levels steadily increased. All blood cultures remained negative. Transaminase levels, which initially declined slightly, began to rise again on Day 8 of admission. Given the ongoing inflammation and elevated transaminase levels, the likely cause was identified as immune-related hepatitis. Antibiotic therapy was discontinued, and methylprednisolone (1 mg/kg/day) was initiated. Her clinical condition improved rapidly thereafter, and she was discharged home on Day 10. Interestingly, a FDG-PET/CT performed 4 days after discharge revealed a complete metabolic response (Figure 1). Laboratory values improved steadily, and corticosteroid therapy was tapered to a complete stop over the next 6 weeks. Two months after discharge, both serum calcium and transaminase levels remained normal. Treatment with nivolumab was reintroduced in January 2025. FDG-PET/CT in February demonstrated a sustained complete metabolic remission. At the most recent follow-up consultation in May 2025, she was doing well, with laboratory results showing no inflammation and normal serum calcium levels.

## 3. Discussion

In the case presented, the patient experienced a rapid onset of severe hypercalcemia after three cycles of ipilimumab–nivolumab. No bone lesions were identified and PET imaging revealed a metabolic complete response just 2 weeks after admission. These findings make hypercalcemia of malignancy highly unlikely and suggest an immune-related origin of the hypercalcemia.

Several case reports have documented instances of hypercalcemia occurring during treatment with immune checkpoint inhibitors, despite controlled malignancy. Notably, multiple mechanisms can disrupt serum calcium homeostasis in this rare immune-related adverse event.

Deligiorgi et al. reported two patients who developed hypercalcemia 11 and 15 weeks after initiation of nivolumab. In both cases, the cancer had a partial response, and the hypercalcemia occurred concurrently with an immune-related pneumonitis. Laboratory studies in both patients revealed elevated PTHrP levels. Both patients responded well to treatment with prednisolone [6].

Both Newman et al. and Miller et al. described cases in which hypercalcemia occurred during treatment with immune checkpoint inhibitors. The patients had undetectable levels of PTHrP and calcitriol. Diagnostic tests demonstrated adrenocorticotropic hormone (ACTH) and cortisol deficiency secondary to immune-mediated hypophysitis. The hypercalcemia in these patients was considered to be secondary to hypocortisolaemia, which can lead to hypercalcemia through various mechanisms [7, 8].

We found five case reports where elevated calcitriol levels were measured in patients treated with immune checkpoint inhibitors who developed hypercalcemia. Two cases involved ipilimumab–nivolumab, while three were treated with antiprogrammed cell death 1 (PD-1) monotherapy. In one case, there was progression of the underlying oncological disease, while the other four had controlled disease. One patient simultaneously developed new intrathoracic lymphadenopathies and elevated serum ACE levels, with biopsy confirming a sarcoid-like reaction. Another patient had pre-existing biopsy-proven sarcoidosis, suspected to have reactivated. A third case showed hypermetabolic mediastinal and bilateral hilar lymph nodes on FDG-PET/CT, with biopsy only revealing reactive lymphoid cells. The remaining two cases did not exhibit any sarcoid-like features. Four patients received treatment with calcitonin, fluids and bisphosphonates or denosumab. Although serum calcium levels decreased, the reduction was either insufficient or short-lived. In all cases, administration of corticosteroids was necessary to normalise serum calcium levels [9–13].

Sarcoidosis is characterised by a hyperactive T helper-1 (Th-1) immune response, prompting the release of interferon gamma (IFN-y) and other proinflammatory cytokines. These cytokines are the stimulus for macrophage activation and aggregation, ultimately leading to the formation of noncaseating granulomas. Increased interleukin-17 (IL-17), produced by T helper-17 (Th-17) cells, is thought to play an additional role in the formation of granulomas. Both PD-1 and cytotoxic T-lymphocyte-associated protein 4 (CTLA-4) inhibitors can promote Th-1 and Th-17 hyperactive immune responses, facilitating a sarcoid-like reaction [14]. A specific subset of T helper cells, termed Th-17.1, can produce IFN-y and/or IL-17. Th-17.1 cells have been implicated in the pathogenesis of several autoimmune disorders, including sarcoidosis. A recent case series report an interesting association between the presence of an abnormally high number of circulating Th17.1 cells and the onset of sarcoid-like reactions in melanoma patients starting on anti-PD-1 therapy [15].

In granulomatous diseases, such as sarcoidosis, abnormal macrophage activation is known to lead to calcitriol-mediated hypercalcemia in 5%–10% of cases [16]. Sarcoid-like reactions, which can be induced by various drugs including immune checkpoint inhibitors, may also rarely result in hypercalcemia. A systematic review of 91 patients with sarcoid-like reactions during treatment with immune checkpoint inhibitors or BRAF/MEK inhibitors for melanoma found that three developed hypercalcemia [17]. The previously mentioned cases illustrate that calcitriol-mediated hypercalcemia can occur even in the absence of granulomatous features. It has been postulated that PD-1 inhibitors may independently activate a subset of macrophages in cancer patients expressing programmed cell death ligand 1 (PD-L1). This macrophage activation could increase 1-alpha hydroxylase activity, thereby shifting calcium homeostasis toward hypercalcemia through elevated calcitriol levels [9, 11, 18].

In the presented case, common causes of hypercalcemia were carefully excluded. The patient had normal morning cortisol levels, adequate vitamin D, suppressed PTH, and did not take any medication that could affect calcium homeostasis. As previously mentioned, the complete metabolic remission observed on FDG-PET/CT makes the diagnosis of hypercalcemia of malignancy improbable. Although not fully recognised during the admission, it is now clear that a sarcoid-like reaction played a central role in our patient's hypercalcemia.

After exposure to pembrolizumab and dabrafenib–trametinib, the patient developed a biopsy-proven sarcoid-like reaction, which persisted on imaging over the years, with evidence still visible in August 2024 (Figure 1). On Day 3 of admission, newly enlarged mediastinal lymphadenopathies were identified on CT (Figure 2). ACE is often used as a diagnostic tool in sarcoidosis, and when hypercalcemia is present in patients with sarcoidosis, elevated ACE levels are found in nearly all patients [19]. In our patient, the serum ACE level was elevated considerably. Additionally, the patient had elevated transaminase levels, systemic inflammation and fever, all of which responded to the use of corticosteroids. The combination of hypercalcemia, elevated ACE, inflammation and newly enlarged mediastinal lymphadenopathies is consistent with a flare-up of the sarcoid-like reaction, likely provoked by the dual immune checkpoint inhibition. This increase in sarcoid-like activity could have elevated 1-alpha hydroxylase activity, thereby facilitating calcitriol-mediated hypercalcemia. Unfortunately, neither PTHrP nor calcitriol was measured in our case, which leaves this hypothesis unconfirmed. One might wonder if the presumed immune-related hepatitis was also a manifestation of the sarcoid-like reaction, as sarcoidosis is a multisystem disease that can cause elevated liver enzymes in nearly one third of patients [20].

It is possible that the methylprednisolone given as allergy prophylaxis played a role in the normalisation of calcium levels, and the gradual tapering of corticosteroids for the presumed hepatitis may have helped prevent early recurrence of hypercalcemia. However, the delayed diagnosis exposed the patient to unnecessary risks. Earlier initiation of methylprednisolone (1 mg/kg/day) could have led to quicker resolution of hypercalcemia, inflammation and hepatitis, thereby reducing the duration of hospitalisation. We regret that we did not promptly recognise the hypercalcemia as an immune-related adverse event, as we were initially not familiar with this rare complication. We hope publishing this case will lead to better management of similar events in the future.

## 4. Conclusion

Immune checkpoint inhibition-induced hypercalcemia is a rare but significant adverse event, with only a limited number of published case reports. Immune checkpoint inhibitors can induce the production of PTHrP, calcitriol, and may cause hypocortisolaemia, all of which can disrupt calcium homeostasis. Remarkably, calcitriol-mediated hypercalcemia can occur with or without the presence of sarcoid-like features. We present a rare case of severe hypercalcemia as an immune-related adverse event in the context of a sarcoid-like reaction, initially induced by dabrafenib–trametinib and subsequently exacerbated by dual checkpoint inhibition. Through this case, we contribute to the growing body of evidence regarding hypercalcemia as an immune-related adverse event and aim to raise awareness among clinicians of this potential complication. Early recognition is critical for this life-threatening condition, as it can be refractory to conventional therapies and may necessitate corticosteroid therapy.

## Figures and Tables

**Table 1:** Relevant laboratory values before, during and after hospital admission. Day −7 corresponds to the administration of the fourth cycle of ipilimumab–nivolumab. Day 0 marks the day of hospital admission and the initiation of pamidronate (90 mg) and isotonic saline. On Day 2, methylprednisolone 32 mg was administered once. Methylprednisolone (1 mg/kg/day) was initiated on Day 8, and the patient was discharged on Day 10.

**Day of admission**	**−16**	**−7**	**0**	**2**	**5**	**7**	**16**	**40**	**Reference**
CRP (mg/L)	15	21	92	64	110	150	11	1	< 5.0
Calcium (mmol/L)	2.4	2.68	3.6	2.99	2.01	1.82	2.33	2.36	2.20–2.55
Albumin-corrected calcium (mmol/L)		2.79	3.84	3.34	2.4	2.23		2.38	2.20–2.55
Creatinine (mg/dL)	1.09	1.56	1.72	1.62	1.34	1.24	1.26	1.04	0.81–0.95
AST (U/L)		32		180	110	180	88	28	11–34
ALT (U/L)	15		170					45	< 34
Albumin (g/L)		34.7	27.9	22.7	20.6	19.6		38.8	32–46

## Data Availability

The data that support the findings of this study are available from the corresponding author upon reasonable request.
